# Competition between *Phytophthora infestans* Effectors Leads to Increased Aggressiveness on Plants Containing Broad-Spectrum Late Blight Resistance

**DOI:** 10.1371/journal.pone.0010536

**Published:** 2010-05-07

**Authors:** Dennis A. Halterman, Yu Chen, Jiraphan Sopee, Julio Berduo-Sandoval, Amilcar Sánchez-Pérez

**Affiliations:** 1 Department of Plant Pathology, University of Wisconsin-Madison, Madison, Wisconsin, United States of America; 2 United States Department of Agriculture-Agricultural Research Service, Vegetable Crops Research Unit, Madison, Wisconsin, United State of America; 3 Department of Plant Pathology, Kasetsart University, Bangkok, Thailand; 4 Faculty of Agronomy, University of San Carlos of Guatemala, San Carlos, Guatemala; Purdue University, United States of America

## Abstract

**Background:**

The destructive plant disease potato late blight is caused by the oomycete pathogen *Phytophthora infestans* (Mont.) de Bary. This disease has remained particularly problematic despite intensive breeding efforts to integrate resistance into cultivated potato, largely because of the pathogen's ability to quickly evolve to overcome major resistance genes. The *RB* gene, identified in the wild potato species *S. bulbocastanum*, encodes a protein that confers broad-spectrum resistance to most *P. infestans* isolates through its recognition of highly conserved members of the corresponding pathogen effector family IPI-O. *IpiO* is a multigene family of effectors and while the majority of IPI-O proteins are recognized by RB to elicit host resistance, some variants exist that are able to elude detection (e.g. IPI-O4).

**Methods and Findings:**

In the present study, analysis of *ipiO* variants among 40 different *P. infestans* isolates collected from Guatemala, Thailand, and the United States revealed a high degree of complexity within this gene family. Isolate aggressiveness was correlated with increased *ipiO* diversity and especially the presence of the *ipiO4* variant. Furthermore, isolates expressing IPI-O4 overcame RB-mediated resistance in transgenic potato plants even when the resistance-eliciting IPI-O1 variant was present. In support of this finding, we observed that expression of IPI-O4 via *Agrobacterium* blocked recognition of IPI-O1, leading to inactivation of RB-mediated programmed cell death in *Nicotiana benthamiana*.

**Conclusions:**

In this study we definitively demonstrate and provide the first evidence that *P. infestans* can defeat an R protein through inhibition of recognition of the corresponding effector protein.

## Introduction

Late blight, caused by the oomycete pathogen *Phytophthora infestans*, remains one of the most devastating diseases of potato and tomato, even after many decades of resistance breeding efforts. The oomycetes belong to a diverse group of eukaryotic microorganisms that are closely related to brown algae in the Stramenopiles, one of several major eukaryotic kingdoms. Oomycete plant pathogens, such as *P. infestans*, secrete many proteins that are important in virulence on the host [1]–[3]. These proteins, termed effectors, are either introduced into the plant extracellular space, where they interact with extracellular targets or surface receptors, or into the plant cell cytoplasm. Deciphering the molecular function of these effectors is key to understanding how these pathogens cause disease. A primary role of pathogen effectors is to suppress host basal defense responses, allowing the pathogen to grow and reproduce [4], [5]. In most cases, host resistance proteins recognize the presence of a single pathogen effector molecule [4]. Activation of resistance proteins elicits a strong resistance response, which includes expression of defense-related proteins, an oxidative burst, and programmed cell death termed the hypersensitive response (HR). Some effectors can suppress resistance responses mediated by R proteins, allowing the pathogen to cause disease even when a corresponding R protein has been activated [6]–[9].

Data mining and functional assays have been very helpful in identifying putative effectors from the *P. infestans* genome sequence [10], [11]. Using this strategy, the avirulence gene *Avr3a* was identified in *P. infestans* and cloned [12]. The Avr3a protein is recognized by resistance protein R3a from *S. demissum* in the host cytoplasm, triggering plant defense responses. Avr3a is one member of a set of hundreds of *P. infestans* secreted proteins carrying a highly conserved N-terminal motif RXLR (X denotes any amino acid) [12]. Whisson et al. [13] provided convincing evidence that the RXLR motif acts as a host cell-targeting signal that mediates trafficking into host cells.

That fact that host resistance proteins often recognize the presence of pathogen effectors has made it practicable to screen late blight resistant *Solanum* species for recognition of putative *P. infestans* effectors [14]. Using this approach, an RXLR effector *ipiO* was found to induce hypersensitive resistance in the wild potato species *Solanum bulbocastanum*, *S. stoloniferum*, and *S. papita* [14]. *IpiO* is only known to be present in *P. infestans* and the closely related species *P. andina*, *P. ipomoeae*, *P. phaseoli*, and *P. mirabilis* [15]. Expression of *ipiO* is induced *in planta* during the early stage of *P. infestans* infection [16] but no definitive function for *ipiO* within the host cytoplasm has been determined. IPI-O variants have been divided into three classes based on diversity of deduced amino acid sequences [15]. Class I and class II variants of IPI-O, which are found in the majority of *P. infestans* isolates, are recognized by the *S. bulbocastanum* resistance protein RB (or Rpi-blb1) [15], [17], [18]. Interestingly, class III IPI-O variants (e.g. IPI-O4) are not recognized by RB, suggesting that a *P. infestans* isolate containing only this variant would be able to overcome *RB*-mediated resistance [15].

*RB* confers partial foliar resistance to late blight with no effect on plant performance [17]–[19]. Resistance mediated by *RB* has been found to be effective against many diverse *P. infestans* isolates due to the almost ubiquitous presence of the corresponding pathogen effector IPI-O class I and class II variants [15]. This differs from *P. infestans* immunity derived from the wild potato species *S. demissum*, where virulent races of the pathogen have overcome a majority of the *R* genes from this host species likely due to mutation or elimination of the corresponding effector [20]. Based on sequence homology to the previously cloned *RB* gene, *Rpi-stol1* and *Rpi-pta1* were cloned from *S. stoloniferum* and *S. papita*, respectively, and shown to also recognize IPI-O1 and IPI-O2 [14]. Functional homologs of *RB* have also been identified in other phylogenetically distant wild potato species, such as *S. verrucosum* [21], confirming that this gene is likely of ancient origin [18] and suggesting functional conservation of the *RB* gene throughout the evolution of potato.

In this study we utilized 40 isolates of *P. infestans* collected from foliar-infected potatoes in Guatemala, Thailand, and the United States to analyze the diversity of IPI-O. Our results support the hypothesis that the *ipiO* locus is extremely variable between isolates, not only in the presence or absence of specific alleles but also in copy number. We have also found that the class III IPI-O variant IPI-O4 not only eludes detection by RB, but is also capable of inhibiting hypersensitive resistance elicited by the class I variant IPI-O1.

## Results

### *IpiO* variants are present among diverse *P. infestans* isolates

To investigate the diversity of the *ipiO* gene in *P. infestans* populations, we collected *P. infestans* from Central America (Guatemala), South East Asia (Thailand) and North America (United States; Table 1). Amplification of the *ipiO* genes using PCR resulted in products of 456 base pairs, which lack the region encoding the signal sequence but contain DNA encoding the RXLR motif and the remainder of the protein as well as a portion of the 3′ untranslated region. Although sequences were divergent, all *P. infestans* isolates yielded amplicons that encode putative protein sequences of 131 amino acids. As expected, each isolate contained multiple copies of *ipiO*. However, the number of *ipiO* variants and the presence or absence of specific sequences differed. Where significant variability in the number of *ipiO* variants was found, as many as 32 additional clones (for a total of 48) were sequenced to ensure that all possible variants had been identified with 99% probability. Interestingly, no isolates were found with the same complement of *ipiO* gene sequences. Overall, among the 40 *P. infestans* isolates, we obtained 248 unique deduced IPI-O amino acid sequences. Class I variants (IPI-O1 and IPI-O2 related) were found in all the *P. infestans* isolates. The presence of class II variants (IPI-O3 related) was only slightly less consistent, with variants of this gene found in 78% of the isolates. An obvious exception was the US isolates, none of which contain class II variants. Class III variants (IPI-O4 related) were much more rare, and were only found in 7 of the 41 isolates (17%).

**Table 1:** *P. infestans* isolates used for IpiO sequencing.

Isolate Name	Area collected (region, state or province, sub-province, country	Isolate #	Race	Obtained from
19	Patzicia, Chimaltenango, Guatemala		unknown	direct collection, 2007
20	Patzicia, Chimaltenango, Guatemala		unknown	direct collection, 2007
27	Georginas, Quetzaltenango, Guatemala		unknown	direct collection, 2007
34	Georginas, Quetzaltenango, Guatemala		unknown	direct collection, 2007
39	Georginas, Quetzaltenango, Guatemala		unknown	direct collection, 2007
40	Georginas, Quetzaltenango, Guatemala		unknown	direct collection, 2007
44	Concepción 3, Quetzaltenango, Guatemala		unknown	direct collection, 2007
46	Concepción 3, Quetzaltenango, Guatemala		unknown	direct collection, 2007
47	Aguas Amargas, Quetzaltenango, Guatemala		unknown	direct collection, 2007
49	Aguas Amargas, Quetzaltenango, Guatemala		unknown	direct collection, 2007
52	Aguas Amargas, Quetzaltenango, Guatemala		unknown	direct collection, 2007
54	Aguas Amargas, Quetzaltenango, Guatemala		unknown	direct collection, 2007
57	Aguas Amargas, Quetzaltenango, Guatemala		unknown	direct collection, 2007
64	Cobán, Alta Verapaz, Guatemala		unknown	direct collection, 2007
67	Cobán, Alta Verapaz, Guatemala		unknown	direct collection, 2007
68	Cobán, Alta Verapaz, Guatemala		unknown	direct collection, 2007
CMPh0-03	Chiang Mai, Fang, Thailand		unknown	direct collection, 2007
CMPh0-05	Chiang Mai, Fang, Thailand		unknown	direct collection, 2007
CMPh0-07	Chiang Mai, Fang, Thailand		unknown	direct collection, 2007
CMSS1-02	Chiang Mai, San Sai, Chedimaekhrua, Thailand		unknown	direct collection, 2007
CMSS1-04	Chiang Mai, San Sai, Chedimaekhrua, Thailand		unknown	direct collection, 2007
CMSS1-08	Chiang Mai, San Sai, Chedimaekhrua, Thailand		unknown	direct collection, 2007
CMSS2-03	Chiang Mai, San Sai, Mae-Faek-Mai, Thailand		unknown	direct collection, 2007
CMSS2-10	Chiang Mai, San Sai, Mae-Faek-Mai, Thailand		unknown	direct collection, 2007
CMSS2-15	Chiang Mai, San Sai, Mae-Faek-Mai, Thailand		unknown	direct collection, 2007
CMSS3-05	Chiang Mai, San Sai, Nong-Han, Thailand		unknown	direct collection, 2007
CMSS3-15	Chiang Mai, San Sai, Nong-Han, Thailand		unknown	direct collection, 2007
CMSS3-24	Chiang Mai, San Sai, Nong-Han, Thailand		unknown	direct collection, 2007
TKPP1-02	Tak, Phob-Phra, RuamThai-Patana, Thailand		unknown	direct collection, 2007
TKPP1-05	Tak, Phob-Phra, RuamThai-Patana, Thailand		unknown	direct collection, 2007
TKPP1-06	Tak, Phob-Phra, RuamThai-Patana, Thailand		unknown	direct collection, 2007
TKPP1-07	Tak, Phob-Phra, RuamThai-Patana, Thailand		unknown	direct collection, 2007
US1a	United States	US940501	0	W. Fry, Cornell University
US1b	United States	WI 94-1	unknown	W. Stevenson, University of Wisconsin
US11a	United States	S37A1994	unknown	W. Stevenson, University of Wisconsin
US11b	United States	US980008	unknown	B. Baker, USDA/ARS
US8a	United States	US940480	0,1,2,3,4,5,6,7,10,11	B. Baker, USDA/ARS
US8b	United States	US930287	unknown	W. Fry, Cornell University
US8c	United States	693-3	0,1,2,3,4,5,6,7,8,10,11	N. Gudmestad, North Dakota State
US8d	United States	126-C-18	0,1,2,3,4,5,6,7,8,9,10,11	N. Gudmestad, North Dakota State

**Figure 1:**
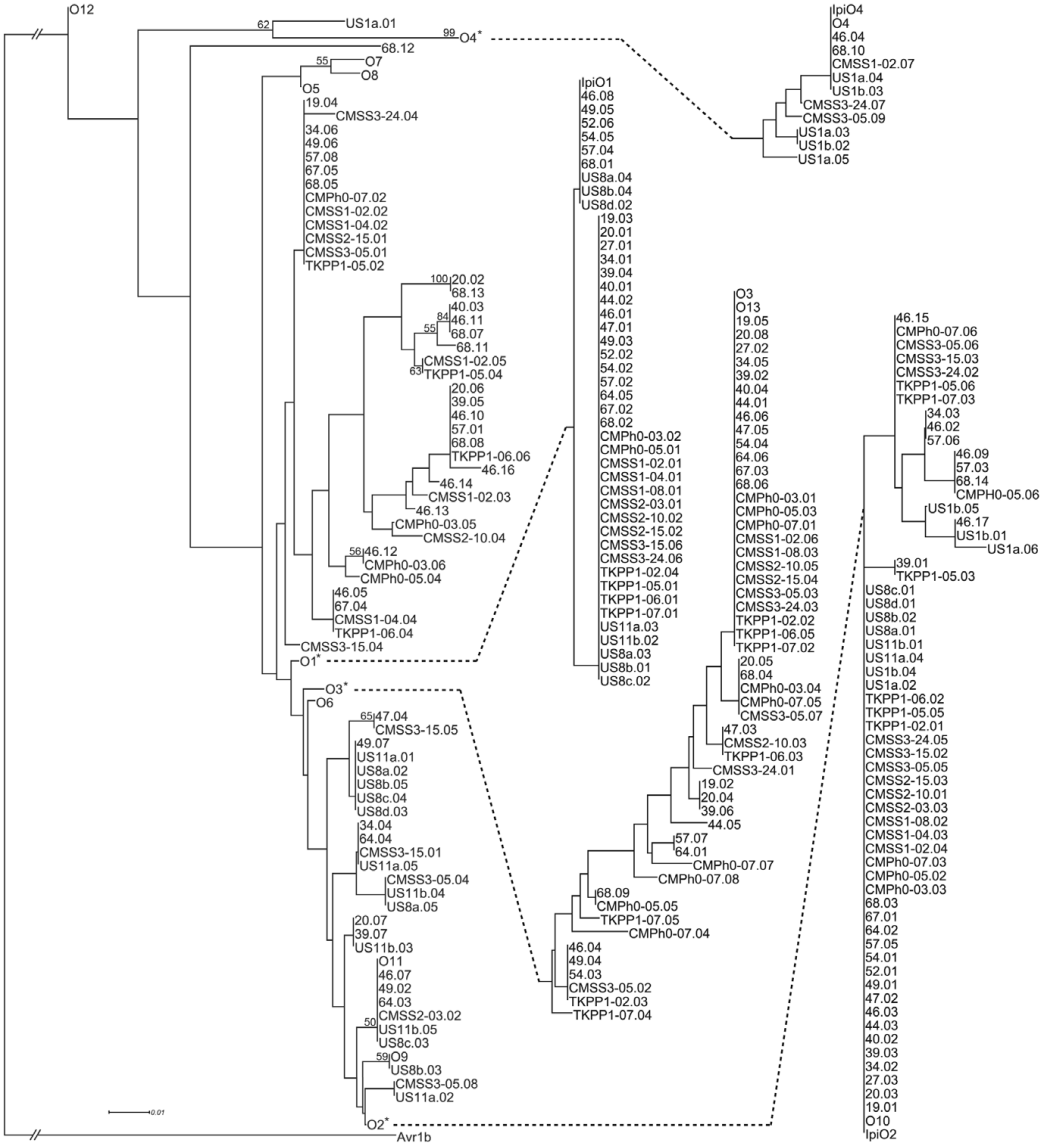
Grouping of IPI-O peptide sequences into multiple clusters. This dendrogram shows hierarchical clustering of deduced amino acid sequences of IPI-O variants identified from 16 *P. infestans* isolates from Guatemala (beginning with numbers), 16 isolates from Thailand (beginning with CMSS, CMPh, or TKPP), and 8 from the United States (beginning with US). Previously identified IPI-O sequences (O1-O13) were also included. The “*” indicates the IPI-O1, IPI-O2, IPI-O3, and IPI-O4 clusters, which are linked to the dendogram by dotted lines. Branch lengths (solid lines) were not altered and represent the evolutionary distances used to deduce the tree. Bootstrap values ≥50% from 1000 replications are shown at the nodes.

Phylogenetic analysis of deduced IPI-O peptide sequences showed a grouping into multiple clusters (Figure 1). The pattern is similar to another recently published IPI-O diversity analysis [15] with organization into three different classes. A cluster of IPI-O4-related sequences (class III) was clearly distinct from the rest of the IPI-O variants. IPI-O3-related variants represent class II and the remaining IPI-O sequences represent class I. Clustering based on nucleotide sequences yielded similar results. Despite the apparent IPI-O diversity present among isolates, only 28 of the 131 amino acids were polymorphic in the sequences derived from our experiments. The majority (23/28) of the polymorphic sites are located in the 80 amino acids C-terminal to the RSLR-EER motif and 12 are located within the 25 amino acid W-motif, a motif sharing homology between diverse *Phytophthora* RXLR-EER effectors [11].

Individual amino acid sites in IPI-O were analyzed for diversifying selection using phylogenetic analysis by maximum likelihood (PAML) [22], [23]. Selection models M2a and M8 both identified the same 14 positively selected amino acids among unique IPI-O sequences (Table 2). A likelihood ratio test of the results of these two models indicated a significant probability that these sites are under positive selection and are not the result of relaxation of selection pressure (*p*<0.001). Interestingly, six of these sites reside within the conserved W-motif (Figure S1). Of the seven amino acid sites previously identified to be under divergent selection among *P. infestans* IPI-Os [15], five overlap with the sites identified in our analysis.

**Table 2:** IPI-O amino acid sites under diversifying selection.

Model	Parameter estimates	lnL^a^	Positively selected sites^b^
M1a	ω0 = 0, ω1 = 1, p0 = 0.749, p1 = 0.251	−1368.65	neutral selection model
M2a	ω0 = 0, ω1 = 1, ω2 = 12.141, p0 = 0.674, p1 = 0.200, p2 = 0.125	−1313.38	30V*, 32Y*, 46N*, 68S*, 87L*, 89G*, 92L*, 113A*, 117S*, 122R*, 124L*, 129L, 134A*, 135S, 143N*
M7	p = 0.111, q = 0.039	−1369.92	neutral selection model
M8	p0 = 0.874, p1 = 0.006, q = 0.226, ω = 11.682	−1313.60	30V*, 32Y*, 46N*, 68S*, 87L*, 89G*, 92L*, 113A*, 117S*, 122R*, 124L*, 129L, 134A*, 135S, 143N*

a log likelihood value.

b *p*<0.05.

* =  *p*<0.01.

### *P. infestans* isolates contain differing numbers of IPI-O variants

The mean/median number of unique deduced amino acid sequences (IPI-O variants) per isolate was 6.8/5.5 in the Guatemalan isolates, 5.6/6.0 in the Thai isolates, and 4.8/5.0 in the U.S. isolates (Table 3). However, in two Guatemalan isolates, named #46 and #68, we found 17 and 14 IPI-O variants, respectively. This level of complexity was not observed in any other isolates although one Thai isolate, CMSS3-05, contained 9 IPI-O variants. In contrast, several isolates, including representatives from Guatemala, Thailand, and the US, contained only 3 IPI-O variants. This variability between isolates is likely not due to amplification-based anomalies since a high-fidelity, high-processivity polymerase and adequate elongation times were used to avoid the possibility of chimera formation during PCR. Additionally, amplification of a majority of the DNA samples produced relatively few variants indicating true copy number variability within the *ipiO* locus.

**Table 3:** Number and classification of IPI-O variants in each *P. infestans* isolate.

			Class						Class						Class		
	Isolate	Unique aa sequences	I	II	III		Isolate	Unique aa sequences	I	II	III		Isolate	Unique aa sequences	I	II	III
Guatemala	19	5	3	2	0	Thailand	CMPh0-03	6	4	2	0	United States	US1a	6	2	0	4
	29	8	5	3	0		CMPh0-05	6	4	2	0		US1b	5	3	0	2
	27	3	2	1	0		CMPh0-07	8	3	5	0		US11a	5	5	0	0
	34	6	5	1	0		CMSS1-02	7	5	1	1		US11b	5	5	0	0
	39	7	5	2	0		CMSS1-04	4	4	0	0		US8a	5	5	0	0
	40	4	3	1	0		CMSS1-08	3	2	1	0		US8b	5	5	0	0
	44	5	2	3	0		CMSS2-03	3	3	0	0		US8c	4	4	0	0
	46	17	15	1	1		CMSS2-10	5	3	2	0		US8d	3	3	0	0
	47	5	3	2	0		CMSS2-15	4	3	1	0		Average	4.8			
	49	7	6	1	0		CMSS3-05	9	5	3	1		Median	5.0			
	52	3	3	0	0		CMSS3-15	6	6	0	0						
	54	5	3	2	0		CMSS3-24	7	4	2	1						
	57	8	7	1	0		TKPP1-02	4	2	2	0						
	64	6	4	2	0		TKPP1-05	6	6	0	0						
	67	5	4	1	0		TKPP1-06	6	4	2	0				Class		
	68	14	10	3	1		TKPP1-07	5	3	2	0			Unique aa sequences	I	II	III
	Average	6.8					Average	5.6				All isolates	Average	5.9	4.3	1.3	0.3
	Median	5.5					Median	6.0					Median	5.0	4.0	1.0	0.0

However, in order to further verify differences in *ipiO* copy number, we performed real-time quantitative PCR using genomic DNA from Guatemalan *P. infestans* isolates #27, #46, #52, and #68 as well as US isolate US8a. Copy number of *ipiO* was determined through comparison to the *P. infestans* single copy nuclear gene *β-tubulin* [24]. Standard curves using RD6F/RD6R and TUB901/TUB1401 primer pairs resulted in correlation coefficients of 0.997 and 0.990, respectively. Amplification efficiency of *ipiO* was 87.1% and *β-tubulin* was 86.8% using these primer pairs. The efficiencies were sufficiently similar to compare threshold cycle differences to determine gene copy number. The 2^ΔΔCt^ method was employed using isolate #27 as a calibrator (with 3 copies of *IpiO*). Using this calibration we estimated that isolate #52 contains 3.5 copies of *ipiO*, #46 contains 13.4 copies, #68 contains 11.3 copies, and US8a contains 5.4 copies using this method. The correlation coefficient between the number of unique amino acid sequences and the estimated copy number determined by RT-PCR was 0.999.

When comparing variant composition between isolates, we observed a correlation between IPI-O variability and the presence of the class II variant IPI-O4 in Guatemalan and Thai isolates (r = 0.69; *p*<0.001). Isolates #46 and #68 were the only two Guatemalan isolates containing IPI-O4. Additionally, the presence of IPI-O4 was correlated with increased IPI-O variability in Thai isolates CMSS1-02, CMSS3-05, and CMSS3-24. Among the US isolates, IPI-O4 was found in both US1 strains, both of which contain above average numbers of IPI-O variants.

### IPI-O variant diversity correlates with pathogen aggressiveness

**Figure 2:**
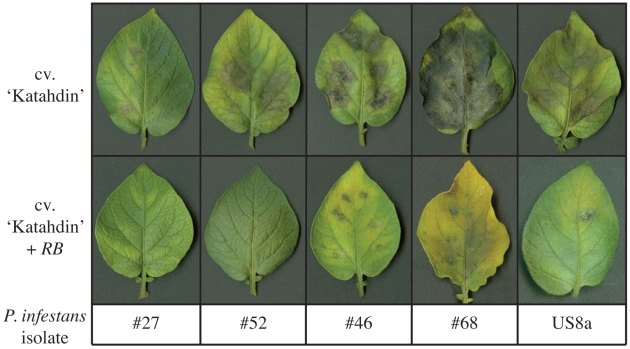
Inoculation of detached leaflets with selected *P. infestans* isolates. Detached leaflets were inoculated with *P. infestans* by placing a 10 µl drop of inoculum at 6 spots on the abaxial surface the leaflets. Photos were taken 5 days after inoculation. Average lesion (necrosis+chlorosis) diameters (in millimeters) of 24 replications are shown in Table 3. Top row: susceptible *S. tuberosum* cv. ‘Katahdin’; bottom row: transgenic ‘Katahdin’ containing the *RB* gene.

In order to determine whether *ipiO* genetic variation correlates with isolate aggressiveness, we used detached leaflets of susceptible potato cv. ‘Katahdin’ and resistant cv. ‘Katahdin’ plants containing a single copy of the *RB* gene (‘SP951’) for inoculation with Guatemalan isolates #46, #68, #27, and #52 along with US8a strain US940480 (5 IPI-O variants; Figure 2; Table 4). Our data show that, based on lesion size, isolate #68, but not #46, was significantly (*p*<0.01) more aggressive on leaves from cv. ‘Katahdin’ compared to the other isolates. Interestingly, isolates with *ipiO4* and multiple variants of other *ipiO* genes (#46 and #68) were significantly (*p*<0.01) more aggressive on plants containing the *RB* resistance gene, indicating their ability to overcome the partial *RB*-mediated resistance response more effectively than those with fewer alleles and no *ipiO4* (#27, #52, and US8a). A comparison of ‘Katahdin’ and ‘SP951’ plants revealed that the presence of the *RB* gene significantly decreased lesion sizes when inoculated with isolates US8a, #52, and #68. The percentage reduction of lesion area due to the presence of the *RB* gene was highest with the US8a and #52 isolates. *RB* had the least effect on isolate #46. This suggested variability among isolates in their ability to overcome *RB*-mediated resistance.

**Table 4:** Results of detached leaflet infection assays.

	Average lesion area^a^		
*P. infestans* isolate	cv. ‘Katahdin’	‘SP951’	% reduction due to *RB*
US8a	62.8±10.7 AY	1.8±1.1 AZ	97.1±1.8%
#27	41.8±13.4 AY	16.6±7.6 AY	60.3±22.2%
#52	69.1±13.1 AY	1.9±1.8 AZ	97.3±2.7%
#46	108.8±15.4 AY	76.4±17.7 BY	29.8±18.3%
#68	388.8±59.1 BY	67.0±14.1 BZ	82.8±4.1%

a lesion areas were calculated by averaging 24 inoculation events. Areas followed by different letters indicate they are significantly different within host genotypes (A and B) or between host genotypes (Y and Z) at *p*<0.01.

### IPI-O4 suppresses IPI-O1-induced HR in the presence of RB

**Figure 3:**
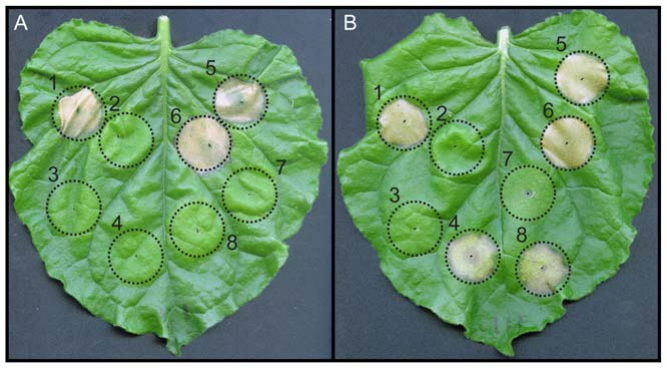
Effect of *ipiO4* on the *ipiO1*-induced hypersensitive response. Non-transgenic (A) and transgenic *N. benthamiana* containing the *RB* gene (B) were infiltrated with *A. tumefaciens* containing the following constructs: 1) pGR106-*INF1*; 2) pGR106-*GFP*; 3) pGR106-*ipiO4*; 4) pGR106-*ipiO1*; 5) pGR106-*INF1* + pGR106-*ipiO4*; 6) pGR106-*INF1* + pGR106-*GFP*; 7) pGR106-*ipiO1* + pGR106-*ipiO4*; 8) pGR106-*ipiO1* + pGR106-*GFP*. Co-infiltration was accomplished using a mixture of equal amounts of *Agrobacterium*. The photograph, which is representative of multiple replications, was taken 7 days after agroinfiltration.

**Figure 4:**
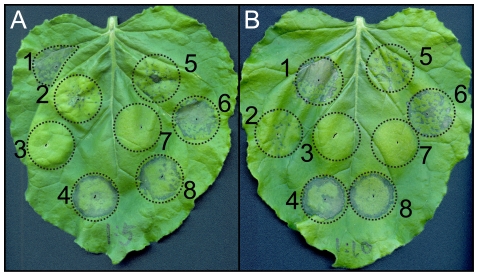
Effect of IPI-O1 overabundance on IPI-O4 mediated HR suppression. Transgenic *N. benthamiana* containing the *RB* gene was infiltrated with *A. tumefaciens* containing the following constructs: 1) pGR106-*INF1*; 2) pGR106-*GFP*; 3) pGR106-*ipiO4*; 4) pGR106-*ipiO1*; 5) pGR106-*INF1* + pGR106-*ipiO4*; 6) pGR106-*INF1* + pGR106-*GFP*; 7) pGR106-*ipiO1* + pGR106-*ipiO4*; 8) pGR106-*ipiO1* + pGR106-*GFP*. Co-infiltration was accomplished using a mixture of *Agrobacterium* strains. **A** contains 5-fold more and **B** contains 10-fold more *Agrobacterium* expressing *INF1* or *ipiO1* in 5, 6, 7, and 8 compared to the co-infiltrated construct. The photograph was taken 7 days after agroinfiltration.

The ability of some isolates to overcome RB-mediated resistance raised the possibility that specific IPI-O variants are able to suppress responses induced by activation of RB. In order to test this, we infiltrated *RB*-transgenic *N. benthamiana* leaves with *A. tumefaciens* strains carrying constructs expressing IPI-O4, IPI-O1, INF1, and green fluorescent protein (GFP; Figure 3). INF1 is a *P. infestans* elicitin that induces an HR in potato and *N. benthamiana* [25]. Additionally, IPI-O1, INF1 and GFP expressing strains were co-infiltrated with the IPI-O4 expressing strain. Hypersensitive cell death was observed 7 days after infiltration in regions infiltrated with *A. tumefaciens* expressing INF1 and IPI-O1 alone. No HR was observed in regions exposed to GFP or IPI-O4. Additionally, no HR was observed in areas co-infiltrated with *A. tumefaciens* expressing IPI-O1 and IPI-O4, indicating a suppression of cell death in the presence of IPI-O4. An HR was observed when IPI-O4 and INF1 were coexpressed, suggesting that IPI-O4 suppression of cell death is IPI-O1-specific. Suppression of the IPI-O1-induced HR was not observed in areas where IPI-O1 and GFP were coexpressed. The suppression phenotype was also observed in overlapping agroinfiltrated regions where IPI-O4-expressing *A. tumefaciens* was infiltrated three days prior to infiltration with bacteria expressing IPI-O1 (Figure S2). In order to test whether an overabundance of IPI-O1 could overwhelm IPI-O4 suppression, *RB*-transgenic *N. benthamiana* plants were infiltrated with a mixture of *A. tumefaciens* where the ratio of bacteria expressing IPI-O4 to those expressing IPI-O1 was 1∶5 and 1∶10 (Figure 4). Despite the increased amount of *A. tumefaciens* expressing IPI-O1, no cell death was observed, demonstrating that IPI-O4-mediated suppression of IPI-O1 recognition can take place even with an expected overabundance of the elicitor.

## Discussion

Late blight remains a worldwide problem for potato production. *P. infestans* is a particularly destructive pathogen and can lead to significant crop losses due to early defoliation and tuber infection. The pathogen spreads rapidly and can cause total crop loss within days [20]. Race-specific resistance to late blight introgressed from *S. demissum* has consistently been overcome by new strains of the pathogen. The *RB* gene, from *S. bulbocastanum*, confers resistance to most isolates of *P. infestans* and has therefore been defined as broad-spectrum [17], [18]. The foliar resistance phenotype mediated by *RB* differs from race-specific immunity derived from the wild species *S. demissum* since *RB*-containing plants allow *P. infestans* to grow and sporulate to a small degree [19], [26]. Separately, we have shown that *RB* is capable of eliciting the HR and other resistance responses, indicating recognition of *P. infestans* effectors (Y. Chen and D. Halterman, submitted). However, *RB*-expressing plants are compromised in their ability to completely stop pathogen spread suggesting that *P. infestans* is able to suppress some RB-mediated responses before the pathogen can be contained. Recently, Champouret and colleagues [15] categorized IPI-O variants into three classes and concluded that the presence of class I variants determine avirulence on plants with *RB* and that *P. infestans* isolates lacking this class are able to overcome resistance mediated by this gene. Our results support these findings and also indicate that the presence of class III variants (IPI-O4) allows *P. infestans* to overcome the avirulence phenotype even when a class I IPI-O is present. It remains unclear, however, whether IPI-O4 interferes with recognition of products of IPI-O expression or elicitation of the HR after recognition by RB of products of IPI-O1 expression. Preliminary evidence suggests a physical interaction between IPI-O4 and a portion of the RB protein (Z. Liu, Y. Chen, and D. Halterman, unpublished data). Interestingly, we have detected no similar interaction between IPI-O1 and RB, suggesting that IPI-O4 may be directly blocking recognition of IPI-O1 rather than affecting the expression, import, or stability of IPI-O1. The fact that IPI-O1 and IPI-O4 were introduced directly into the cell by *Agrobacterium* further suggests that inhibition is taking place in the cytoplasm, where RB is presumably located. Whether IPI-O4 is able to inhibit recognition of other class I or II IPI-O variants has yet to be determined and will be a focus of future research.

The ability of IPI-O4 to inhibit programmed cell death mediated by RB is not unique to the co-evolution of *P. infestans* and its hosts. The *I* gene from the flax rust pathogen *Melampsora lini* empowers a normally avirulent strain with the ability to overcome resistance conferred by specific *R* genes [7], [27], [28]. This activity likely acts at the protein level since the pathogen is able to express the corresponding effector genes normally. Data accumulated from the *I* effect suggest that inhibition of the R protein is controlled via interactions between different domains within the protein and determined by sequence variation in interacting regions [7], [27], [28]. Similarly, the ATR1^NdWsB^ RXLR effector from the oomycete pathogen *Hyaloperonospora parasitica* elicits a resistance response mediated by the corresponding Arabidopsis *R* gene *RPP1-WsB* when expressed with the *R* gene via cobombardment [9]. However, the Emco5 isolate containing this effector is virulent on plants with *RPP1-WsB* [9]. Although the mechanisms involved in allowing Emco5 to overcome resistance are unknown, the authors suggest the possibility that Emco5 is able to evade or suppress recognition of ATR1^NdWsB^ by RPP1-WsB [9]. Effectors of the bacterial pathogen *Pseudomonas syringae* pv. DC3000 also function to suppress resistance in tomato and Arabidopsis. The *Fen* gene of tomato is in the same gene family as *Pto*, which encodes a protein kinase and confers resistance to *P. syringae* expressing the effectors *AvrPto* and/or *AvrPtoB* [29], [30]. Despite 80% amino acid identity, Fen is not able to elicit resistance in the presence of AvrPto or AvrPtoB [31]. Molecular analysis of AvrPtoB has revealed that it encodes a ubiquitin ligase and that Fen is able to elicit resistance to a truncated version of AvrPtoB lacking ligase activity [6], [32]. Full-length AvrPtoB is able to suppress Fen-mediated immunity through ubiquitination of Fen itself leading to degradation of the protein, which demonstrates evolution of the effector to overcome *R* gene-mediated resistance [6]. Similarly, the *P. syringae* effector AvrRpt2 disrupts signaling by the resistance protein RPM1 in Arabidopsis by cleaving an essential RPM1-interacting protein, RIN4 [8]. The mechanism by which the *P. infestans* effector IPI-O4 is able to suppress RB-mediated resistance remains to be fully elucidated. However, results from the work presented here suggest that the phenomenon is specific for RB and that IPI-O4 is not a general suppressor of cell death. It will be interesting to determine whether wild potato species that are able to recognize IPI-O4 to elicit resistance (e.g. *S. stoloniferum*) contain “defeated” *RB*-like genes within the locus that allow for recognition of a different suite of IPI-O variants, or whether other factors are involved in IPI-O4 recognition.

Expression of *RB* is closely correlated with the resistance phenotype and transgenic plants with multiple integration events are typically more resistant than those with fewer copies of *RB* [33], [34]. ‘SP951’, the transgenic potato plants used in our detached leaflet assays, is transgenic *S. tuberosum* cv. ‘Katahdin’ containing a single copy of the *RB* gene. Isolates containing IPI-O4 appeared more aggressive, even when RB was present, possibly due in part to inhibition of IPI-O1 recognition. Interestingly, Guatemalan isolate #27, which does not appear to contain *ipiO4*, was able to overcome RB-mediated resistance at a similar relative level as isolate #68. This suggests that other factors may be involved, such as *ipiO* expression or interaction with unidentified host- or pathogen-derived factors. It is possible that an increase in RB expression could overcome IPI-O suppression and restore the resistance phenotype, although further testing of transgenic plants with increased levels of RB expression is necessary to make this determination. The ability to manipulate resistance in this fashion may be completely dependent on the number of IPI-O4 molecules that enter the plant cell and whether RB expression can be increased enough to overcome suppression of IPI-O1 recognition.

The majority of IPI-O diversity in the isolates analyzed in our experiments was restricted to class I, although nearly all isolates also contained at least one class II variant. The median number of total IPI-O variants from all of the isolates was 5, and most of those variants belong to class I, with a median number of 4. However there were obvious exceptions, with Guatemalan isolates #46 and #68 containing 17 and 14 IPI-O variants, respectively. It is unclear at this point what may have driven diversity at the *ipiO* locus in these isolates. The two strains were collected from separate locations, and other isolates taken from similar locations did not have this level of IPI-O diversity. Isolates #46 and #68 were collected from *S. tuberosum* plants under field conditions suggesting that the increase in IPI-O diversity is not necessarily due to an interaction with resistant hosts. However, *S. bulbocastanum* and *S. demissum*, both of which contain major late blight resistance genes, are present in Guatemala in regions near where the isolates were collected [35]. It is therefore possible that these isolates have had recent contact with resistant or partially resistant wild species, leading to a dramatic increase in IPI-O diversity. This does not explain the increased number of IPI-O variants in some Thai isolates since wild potato species are not common in Thailand. This would suggest that other unknown factors might also influence IPI-O diversity. Previously, two other *P. infestans* isolates exhibiting virulence on *RB*-containing plants, PIC99189 and PIC99177, were collected from the wild species *S. stoloniferum*, from which a functional *RB* homolog has been identified [15], [36], [37]. However, in the case of PIC99189 and PIC99177, virulence was attributed to the absence of class I IPI-O variants [15], whereas we have found that the presence of certain IPI-O variants, namely IPI-O4, may impact an isolate's ability to overcome resistance.

Based on previous published data [15], [38], we expected to find relatively few IPI-O variants. We were surprised by the level of diversity present within the *P. infestans* isolates used in this study, particularly those collected recently from infected potato leaves. Incomplete primer extension during the PCR elongation step can result in chimera formation *in vitro* during the amplification of these products [39]. However, preventative measures (adequate elongation time and high-fidelity polymerase) were used to minimize this possibility. The possibility also exists that IPI-O diversity is maintained in wild populations of *P. infestans* and may be lost after removal of the pathogen from its natural habitat. This could explain why the majority of Guatemalan and Thai isolates contain increased IPI-O diversity. The US isolates used in this study, which have not been exposed to resistant plants for several years, contained the lowest average and median number of unique IPI-O amino acid sequences. We are currently in the process of passaging isolates through *RB*-containing hosts to determine whether this interaction can affect IPI-O diversity over multiple generations.

Up until the early 1990s, US1 was the most prevalent strain of *P. infestans* in the US, but was replaced by US8 and US6 isolates within a period of a few years [40], [41]. The US1 isolate used in this study has been used previously to test for virulence on *RB*-containing plants with no notable increase in aggressiveness despite the fact that it contains *ipiO4* [17, D. Halterman, unpublished data]. This would suggest that factors contributing to virulence on plants with the *RB* gene remain to be found. Despite the fact that RB confers broad spectrum resistance, isolates that can overcome resistance have already been identified [15]. Therefore, it is essential to identify germplasm with resistance sources that will allow recognition of diverse IPI-O variants. Pyramiding of *R* genes that recognize different *P. infestans* effectors, whether they be multiple variants of *ipiO* or unrelated effectors such as *avrRpi-blb2*, will be important in battling this disease at the breeding level. The *RB* gene remains a valuable trait because it confers broad-spectrum resistance to *P. infestans* with no impact on yield in tested transgenic cultivars [19]. The accumulated costs of control efforts and losses due to late blight alone are estimated at more than $3 billion/year worldwide [20]. Therefore, the deployment of plants that require little or no fungicide input and remain resistant even under conditions ideal for disease development should significantly impact costs associated with growing potato everywhere the crop is grown.

## Materials and Methods

### Collection and maintenance of *P. infestans*

Infected leaf samples were collected from areas of major potato production during the rainy seasons in Guatemala and Thailand (August and September 2007 in Guatemala; February and December in Thailand). Guatemalan and Thai isolates were collected from *S. tuberosum* plants of varying cultivars. Leaves with a single lesion of late blight were collected and the process of isolation was started the same day of collection. Late blight-infected lesions were excised from the leaf and placed on clarified V8 (cV8) agar (15% clarified V8 juice, 1.5% CaCO_3_; and 1.5% agar in distilled water) and incubated in the dark at 15°C. Subsamples of *P. infestans* hyphal tips were collected and placed on fresh cV8 media and stored in the dark at 15°C.

### Isolation of *ipiO* alleles from *Phytophthora*

*P. infestans* isolates were grown in liquid pea broth media and DNA was isolated using a previously published protocol [42]. Fifty nanograms of *Phytophthora* DNA were used as a template for polymerase chain reaction (PCR). Primers RD6F (5′ - CGC**ATCGAT**GGTTTCATCCAATCTCAACACCGCCG - 3′) and RD6R (5′ - GAT**GCGGCCGC**TATACGATGTCATAGCATGACA - 3′) were used to amplify *ipiO* alleles using the following parameters: 94° for 1 min; 40 cycles of 94° for 15 s, 52° for 30 s, 68° for 1.5 min; 68° for 15 minutes. All amplifications were carried out using Platinum® PCR SuperMix High Fidelity (Invitrogen, Carlsbad, CA) with 5 nmoles of each primer. PCR products were cloned into pGEM-T Easy vector (Promega, Madison, WI) according to the manufacturers instructions.

### Sequencing and analysis

At least 16 plasmid clones containing *ipiO* variants from each isolate were sequenced in both directions using vector-specific primers. Poisson distribution was used to ensure that the cumulative probability of finding all alleles was greater than 99%. Double-strand sequencing of DNA was carried out at the University of Wisconsin-Madison Biotechnology Center sequencing facility. Vector sequence removal was performed using Vector NTI software (Invitrogen, CA). Duplicate identical sequences from the same *P. infestans* isolate were removed from the analysis. Sequence alignments were done using ClustalX [43] using the *P. infestans* effector Avr1b as an outgroup. Trees were visualized using Dendroscope [44]. Bootstrap values in percentage (≥50) from 1000 repetitions are shown at the nodes.

### Identification of positively selected sites

The 78 unique *IpiO* DNA sequences were used to identify amino acid codons under divergent selection using the codeml program within the PAML v. 4 package [23]. Maximum-likelihood models M0, M1a, M2a, M7 and M8 were used for the analysis. The log-likelihood values from the M1a (neutral selection) and M2a (positive selection) models and the M7 (neutral selection) and M8 (positive selection models were used to perform a likelihood ratio test (LRT). The LRT statistic was calculated as twice the difference in log-likelihood values for the two models, and the cumulative distribution of test statistics for each set of simulated data was plotted against a chi-square distribution with two degrees of freedom.

### Estimation of *ipiO* copy number using real time PCR

Real-time PCR reactions were carried out using a Bio-Rad MyiQ thermocycler (Bio-Rad, CA). All reactions were run in triplicate. For each reaction, 1 µl of each primer and 50 ng of *P. infestans* genomic DNA was added to 22.5 µl of iQ SYBR Green Supermix (Invitrogen, CA). Primers RD6F and RD6R were used to amplify *ipiO* and primers TUB901 (5′-TACGACATTTGCTTCCG-3′) and TUB1401 (5′- CGCTTGAACATCTCCTGG-3′) were used to amplify *β-tubulin* [24]. PCR was performed as follows: 3 min. at 94°C; 40 cycles of 15 sec at 94°C, 30 sec at 56°C, and 30 sec at 68°C. Reactions were followed by melt curve analysis to verify the presence of only one amplicon. The Ct values were determined using the instrument's software. We calculated *ipiO* copy number using the 2^ΔΔCt^ method [45] where ΔCt = Ct (*ipiO*) - Ct (*β-tubulin*) and ΔΔCt = ΔCt (unknown isolate) - ΔCt (isolate #27). Estimated copy number was determined using the 2^ΔΔCt^ and calibrating the results so that the reference isolate #27 contained three copies of *ipiO*.

### Agroexpression of IPI-O variants in *Nicotiana benthamiana*

*N. benthamiana* plants were transformed with the *RB* gene at the University of Wisconsin-Madison Biotechnology Center plant transformation facility. The *RB* gene construct used for transformation was the same as previously published [17]. Agroexpression of *ipiO* variants was performed using vector pGR106 [46] in *Agrobacterium tumefaciens* strain GV3101 [47] as previously described [48]. *A. tumefaciens* strains were diluted to a concentration of OD_600_ = 0.3 before infiltration with a needleless syringe. Inoculated *N. benthamiana* plants were incubated in a growth chamber (22°C day/18°C night temperatures with 16 h of light). Photographs were taken 7 days after inoculation.

### Detached leaf infection assays

All potatoes were propagated from cuttings and maintained in the greenhouse, which was set for 18 h of daylight, a daytime temperature between 17 and 19°C, and a nighttime temperature between 13 and 15°C. Leaflets from six to eight week old cv. ‘Katahdin’ and ‘SP951’ (cv. ‘Katahdin’ with one copy of *RB*) plants were collected. Petioles were trimmed and the leaflets were inserted into plastic boxes containing 0.67% water agar 2 to 24 hours before inoculating with 2 leaflets per cube. *P. infestans* cultures (16–22 days old) were flooded with sterile, distilled water and washes were combined to obtain a suspension of approximately 45,000 sporangia/ml. Sporangial suspensions were placed at 12°C for 3 hours to induce zoospore release. Leaflets were then inoculated with 6 evenly spaced 10 µl drops per leaflet, for a total of 24 independent inoculations per isolate/genotype interaction. Inoculations with each *P. infestans* isolate were repeated twice. Cubes were covered and placed in a 15°C incubator for 6 days before reading results. Six days after inoculation, necrotic plus chlorotic lesion diameters were measured in millimeters. The diameters were used to calculate average lesion areas per inoculation from the 24 inoculation events.

## Supporting Information

Figure S1 Amino acid alignment of IPI-O sequences. For simplicity, duplicate sequences were removed. IPI-O1 sequence is shown along the top. Identical amino acids were replaced with “.” while polymorphic amino acids are shown. A “*” denotes amino acids determined to be under selection for divergence (see Table 2). Lines above the sequence show the RXLR/RGD, DEER, and predicted W motifs from left to right, respectively. Secondary structure prediction (shown at bottom) was done using the PSIPRED protein structure prediction server (http://www.psipred.net/psiform.html). C = coil, E = strand, H = helix. Confidence values (0 = low, 9 = high) are shown below each structure prediction.
(0.26 MB DOC)

Figure S2 Transgenic *N. benthamiana* containing the *RB* gene was infiltrated with *A. tumefaciens* containing the following constructs: A), C), and F) pGR106-*IpiO1*; B) and D) pGR106-GFP; E) and G) pGR106-ipiO4. C) and F) were infiltrated three days after the other constructs. The photograph was taken 5 days after agroinfiltration of the final constructs.
(2.29 MB DOC)
