# Supplementary figures and images for: Competition between Phytophthora infestans Effectors Leads to Increased Aggressiveness on Plants Containing Broad-Spectrum Late Blight Resistance
Source: PLoS One. 2010 May 7;5(5):e10536. doi: 10.1371/journal.pone.0010536 (PMC2866322; doi:10.1371/journal.pone.0010536)

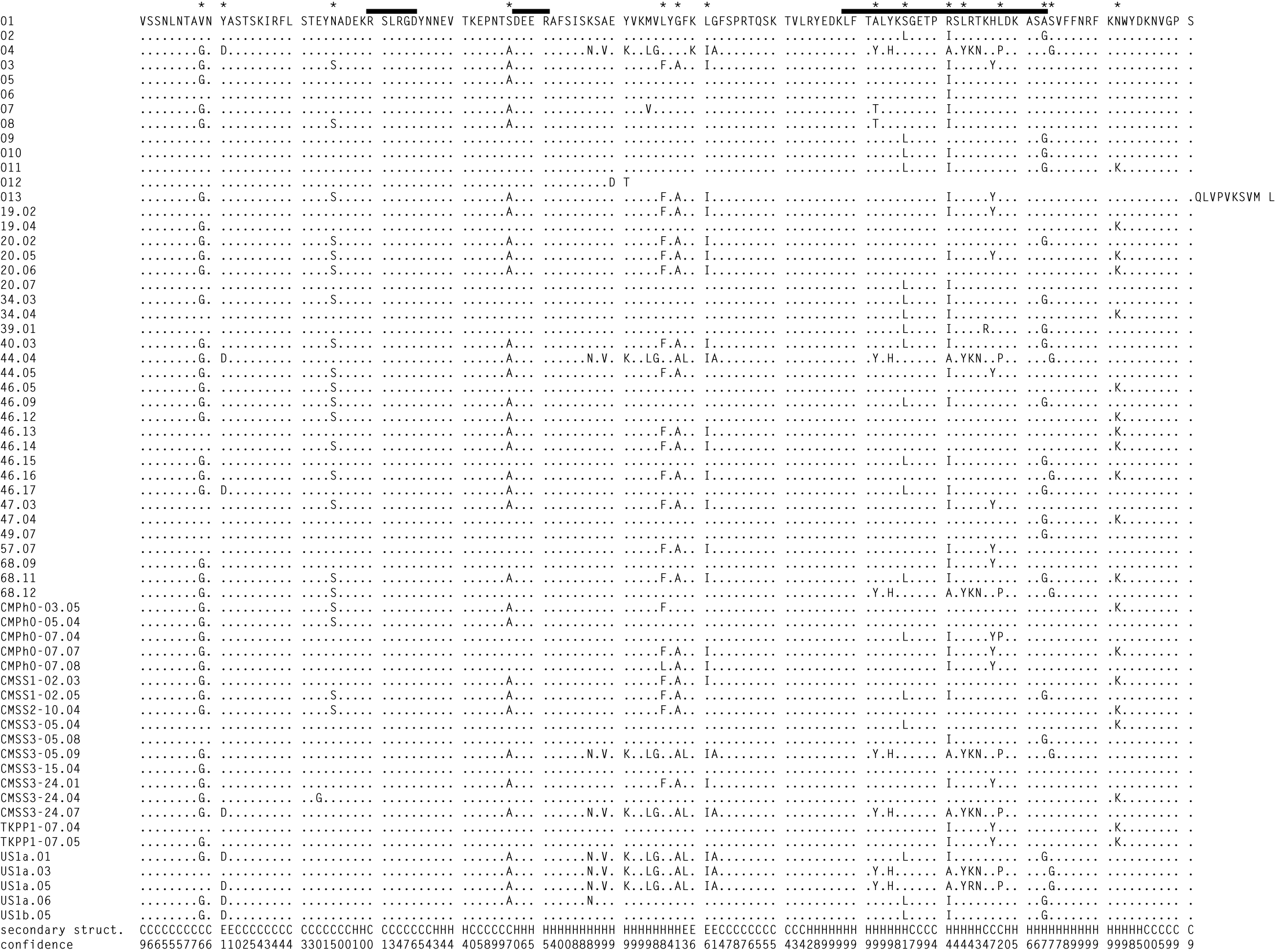

Supplement: Figure S1 — Amino acid alignment of IPI-O sequences. For simplicity, duplicate sequences were removed. IPI-O1 sequence is shown along the top. Identical amino acids were replaced with “.” while polymorphic amino acids are shown. A “*” denotes amino acids determined to be under selection for divergence (see Table 2). Lines above the sequence show the RXLR/RGD, DEER, and predicted W motifs from left to right, respectively. Secondary structure prediction (shown at bottom) was done using the PSIPRED protein structure prediction server (http://www.psipred.net/psiform.html). C = coil, E = strand, H = helix. Confidence values (0 = low, 9 = high) are shown below each structure prediction. (0.26 MB DOC) [file pone.0010536.s001.doc]

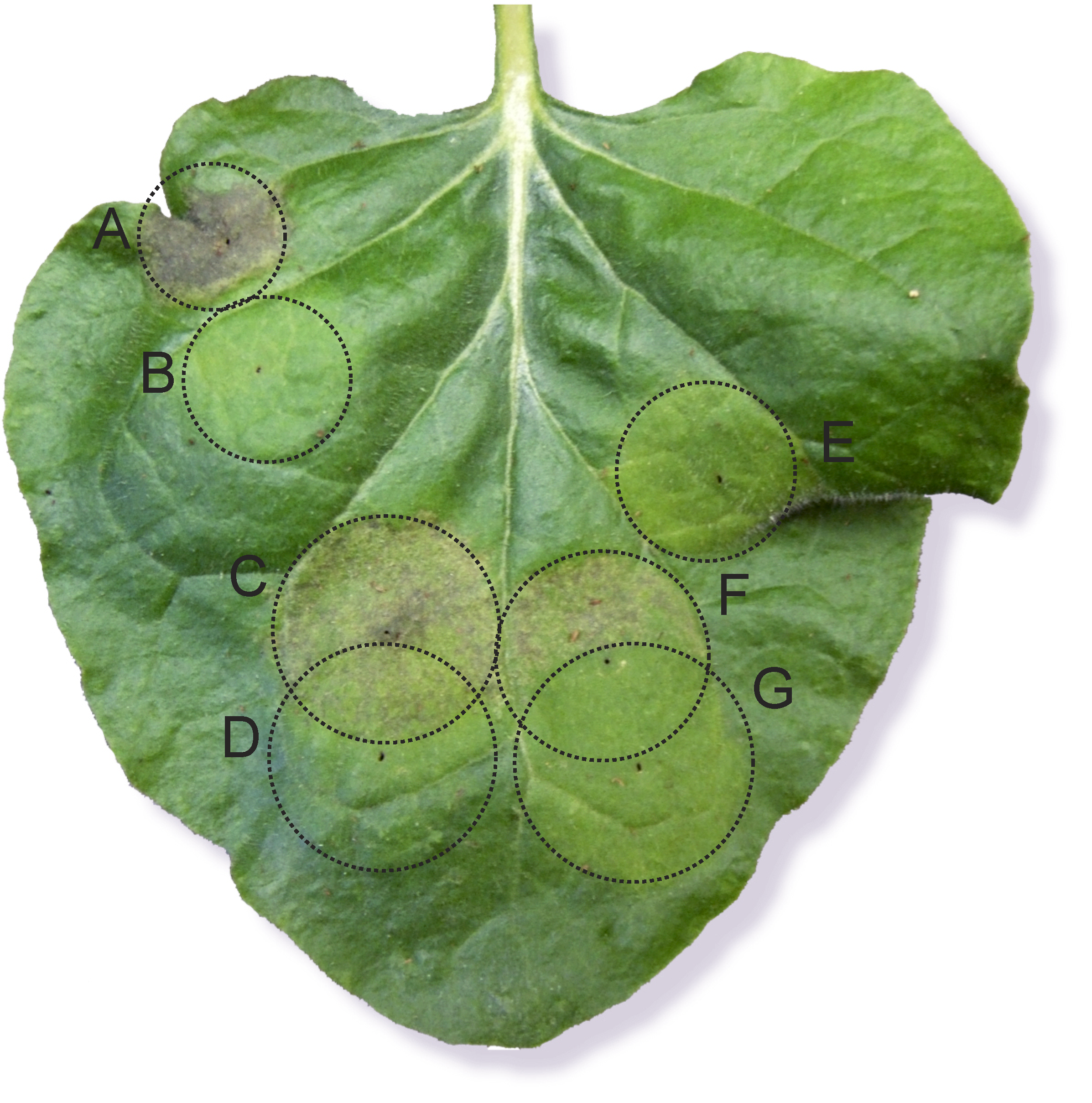

Supplement: Figure S2 — Transgenic N. benthamiana containing the RB gene was infiltrated with A. tumefaciens containing the following constructs: A), C), and F) pGR106-IpiO1; B) and D) pGR106-GFP; E) and G) pGR106-ipiO4. C) and F) were infiltrated three days after the other constructs. The photograph was taken 5 days after agroinfiltration of the final constructs. (2.29 MB DOC) [file pone.0010536.s002.doc]
